# Soil Microbiome Signatures Associated with Epidemiological Heterogeneity Across Natural Plague Foci in Inner Mongolia, China

**DOI:** 10.3390/microorganisms14102329

**Published:** 2026-10-13

**Authors:** Yulong Wang, Jianyun Li, Xudong Yang, Fang Liu, Guangqian Pei, Mengnan Cui, Yan Guo, Yihua Jia, Shengju Zhang, Lili Bao, Yujun Cui, Yong Huang

**Affiliations:** 1 Basic Medical College, Inner Mongolia Medical University, Hohhot 010110, China; wyulong_2000@163.com (Y.W.); zhangshengju24@163.com (S.Z.); baolili1203@126.com (L.B.); 2 State Key Laboratory of Pathogen and Biosecurity, Academy of Military Medical Sciences, Beijing 100071, China; pgqd2008@163.com (G.P.); mengncui@163.com (M.C.); muhan0425@126.com (Y.G.); 3 Inner Mongolia Autonomous Region Center for Disease Control and Prevention, Hohhot 010000, China; hhhtlijianyun@163.com (J.L.); 15149768776@163.com (X.Y.); hhhtliufang@163.com (F.L.); 4 Baotou Medical College, Inner Mongolia University of Science & Technology, Baotou 014000, China; 5 College of Pharmaceutical Sciences, Soochow University, Suzhou 215006, China; 1161897845@163.com

**Keywords:** natural plague foci, metagenomics, soil microbiome, environmental surveillance, bacteriophage

## Abstract

Plague persistence relies on complex host–vector–environment interactions, yet the ecological contribution of soil microbiomes to natural foci remains poorly understood. We performed shotgun metagenomic sequencing of 143 soil samples across four distinct plague foci in Inner Mongolia, China, to characterize microbiome features associated with contrasting epidemiological states. Taxonomic, functional, and network analyses showed that the three active plague foci exhibited lower microbial diversity, simplified co-occurrence networks, and enrichment of specific taxa, particularly *Streptomyces*. Conversely, the quiescent *Marmota sibirica* focus harbored a more complex and interconnected microbial network with greater richness and exclusive detection of *Yersinia* bacteriophages, showing significant concordance with bacterial community structure. These patterns indicate distinct microbiome configurations associated with specific host and geographic settings, highlighting the need to disentangle host- and site-specific effects from broader epidemiological status. Compared with surface and deep soil, burrow-associated soil exhibited greater microbial differentiation and higher richness of bacteriophages, antimicrobial resistance genes, and virulence-associated genes, suggesting its potential as a sensitive environmental matrix for ecological monitoring. Overall, our findings identify distinct soil microbiome signatures—specifically taxonomic shifts, altered network complexity, exclusive *Yersinia* phage detection, and functional gene enrichment—associated with epidemiological heterogeneity across natural plague foci, providing a baseline for understanding ecological variation among endemic habitats.

## 1. Introduction

Plague, caused by *Yersinia pestis*, is one of the most notorious zoonotic diseases in human history and continues to pose a public health threat worldwide [1,2]. Despite substantial reductions in global incidence, natural plague foci remain widely distributed across Asia, Africa, and the Americas, where the pathogen persists through enzootic transmission cycles involving wild rodents and flea vectors [2]. Traditionally, plague ecology has been interpreted within the framework of host–vector–pathogen interactions, with considerable effort devoted to understanding fluctuations in host abundance, flea density, and climatic drivers of disease transmission [3,4]. However, these factors alone cannot fully explain the remarkable spatial and temporal heterogeneity observed among natural plague foci.

Increasing evidence suggests that environmental reservoirs may play a more important role in plague ecology than previously appreciated [5]. Experimental studies have demonstrated that *Y. pestis* can survive in soil for extended periods under favorable conditions and may persist within free-living amoebae or biofilm-like microenvironments [6,7]. These observations imply that soil may function not merely as a transient substrate but as a potential environmental reservoir capable of influencing pathogen persistence and re-emergence [8,9,10,11]. Nevertheless, the ecological context in which *Y. pestis* survives in soil remains largely unknown. In particular, little is understood about how indigenous soil microbial communities influence pathogen persistence, microbial competition, or ecological resilience within natural plague ecosystems.

In China, there are twelve natural plague foci that reflect long-term host adaptation and geographic diversification [12,13,14]. Among them, Inner Mongolia contains four ecologically distinct foci associated with *Meriones unguiculatus*, *Lasiopodomys brandtii*, *Spermophilus dauricus*, and *Marmota sibirica*. These foci exhibit different epidemiological heterogeneity despite their geographical proximity [15]. The *M. unguiculatus* plague focus has remained persistently active over recent decades and accounts for most reported human plague cases in the region [16], whereas the *M. sibirica* plague focus has remained epidemiologically quiescent despite historical evidence of pathogen circulation [17,18]. It is important to clarify that the term “quiescent state” used herein is strictly an epidemiological designation—indicating the prolonged absence of detectable animal epizootics—and does not imply absolute physiological “dormancy” of *Yersinia pestis* or the broader soil microbiome, which likely remain ecologically dynamic. Climatic variation, host population dynamics, or landscape characteristics have been proposed to underlie these differences [19,20]. However, the contribution of belowground microbial ecosystems to this long-term ecological divergence has received little attention.

Traditional targeted surveillance captures only a fraction of the microbial interactions and functional potential within plague foci [2,21]. Consequently, it provides limited information on the soil microbial communities, functional genetic elements, and microbial antagonists that may be associated with the long-term persistence or quiescence of natural plague foci. To overcome this monitoring bottleneck, shotgun metagenomic sequencing, owing to its culture-independent, unbiased capture of environmental genetic information [22,23], has emerged as a pivotal tool for uncovering cryptic pathogens and disentangling complex ecological interaction networks [24].

Here, we used shotgun metagenomic sequencing to profile 143 soil samples collected from four natural plague foci in Inner Mongolia. A standardized multi-scale sampling design included distinct host habitats, soil depths, and burrow-associated microenvironments. We compared microbial community composition, functional gene repertoires, and bacteriophage profiles across plague foci exhibiting distinct epidemiological states. We further explored microbial ecological features potentially associated with long-term persistence or quiescence of plague foci and evaluated different soil matrices for their suitability in environmental surveillance. By integrating taxonomic, functional, and bacteriophage evidence, this study provides new ecological insights into the environmental dimension of plague maintenance and expands the conceptual framework for microbiome-informed surveillance of natural plague foci.

## 2. Materials and Methods

### 2.1. Soil Sampling Strategy and Sample Preparation

Between September 2024 and May 2025, we collected 143 soil samples from epidemiologically active and quiescent natural plague foci in Inner Mongolia, China (Table S1). The a priori classification of these foci as active or quiescent was based on continuous epidemiological surveillance data provided by the local Centers for Disease Control and Prevention (CDC). Specifically, ‘active’ foci were defined as regions with documented animal plague epizootics or *Yersinia pestis* isolation from animal hosts or vectors within recent years. In contrast, the ‘quiescent’ focus was defined as a historically endemic region where no active animal plague epizootics have been detected for over a decade despite routine surveillance. Sampling targeted the habitats of four primary hosts: *Meriones unguiculatus*, *Lasiopodomys brandtii*, *Spermophilus dauricus*, and *Marmota sibirica*, spanning seven counties (Otog Banner, Siziwang Banner, Erenhot City, Sonid Left Banner, Abag Banner, Zhengxiangbai Banner, and Chen Barag Banner). We employed a standardized, depth-stratified sampling strategy. Surface soil (15–30 cm depth) was collected using a five-point sampling design arranged in a quincunx pattern and pooled into a composite sample. Deep soil (1 m ± 5 cm depth) was obtained via vertical coring at the geometric center of each predefined sampling quadrat. Inside-burrow soil samples were collected from host burrows at approximately 30 ± 5 cm depth using standardized spatial and volumetric criteria. Outside-burrow soil samples were collected from the surface soil approximately 1 m away from the burrow entrance following the same sampling protocol. All samples were immediately passed through a sterile sieve to remove plant roots, stones, and macroscopic debris. Processed samples were sealed in sterile bags, transported under cold-chain conditions, temporarily stored at −20 °C, and subsequently transferred to −80 °C until DNA extraction following established protocols [25,26,27,28].

### 2.2. DNA Extraction and Shotgun Metagenomic Sequencing

Total genomic DNA was extracted from soil samples using the DNeasy PowerSoil Pro Kit (Qiagen, **Hilden**, Germany). DNA concentration and purity were assessed using a Qubit fluorometer(Thermo Fisher Scientific, Waltham, MA, USA) and a NanoDrop spectrophotometer (Thermo Fisher Scientific, Waltham, MA, USA), respectively, and DNA integrity was evaluated by 1% agarose gel electrophoresis. Shotgun metagenomic libraries were constructed from fragmented DNA and sequenced on an Illumina NovaSeq 6000 platform (Illumina, San Diego, CA, USA) to generate 150-bp paired-end (PE150) reads. DNA extraction, library preparation, and sequencing were performed by Shanghai Majorbio Bio-Pharm Technology Co., Ltd. (Shanghai, China).

### 2.3. Shotgun Metagenomic Data Processing and Functional Profiling

Raw paired-end FASTQ reads were quality-filtered using Trimmomatic (v0.38) [29] to remove adapter contamination and low-quality sequences (parameters: SLIDINGWINDOW:4:15, MINLEN:40). High-quality reads were taxonomically classified using KrakenUniq (v1.0.4) [30] against a KrakenUniq database (https://benlangmead.github.io/aws-indexes/k2, accessed on 12 July 2023). To accurately represent the compositional structure of the known microbial community, normalized relative abundances were computed by dividing the read counts assigned to a specific taxonomic node by the total number of classified reads in each sample, thereby excluding unmapped or unclassified reads from the compositional baseline. To evaluate the accuracy of KrakenUniq assignments, representative taxa spanning different abundance levels (*Kibdelosporangium phytohabitans*, *Nitrosospira briensis* C-128, and *Planomicrobium glaciei*) were independently validated. Quality-filtered reads were mapped against the corresponding reference genomes using BWA-MEM (v0.7.18) [31] (parameters: -k 31, -M, -T 75), and alignments were filtered using SAMtools(v1.18) by retaining reads with MAPQ > 20 and excluding secondary and supplementary alignments (-F 2308). Functional profiles of antimicrobial resistance genes, virulence factors, and bacteriophage sequences were generated by aligning sequences against the ResFinder (https://bitbucket.org/genomicepidemiology/resfinder_db/src/master, accessed on 6 May 2025), VFDB (http://www.mgc.ac.cn/VFs/download.htm, accessed on 6 May 2025), and PhageFinder (https://phagescope.deepomics.org/download, accessed on 17 July 2025) databases, respectively, using BLAST+ (v2.16.0+) [32]. Alignments were filtered using stringent criteria (E-value < 1× 10^−5^, nucleotide identity ≥ 90%, and query coverage ≥ 80%), and only the top hit per query was retained. Candidate *Y. pestis*-associated phage sequences were further mapped to the reference genome (NC_055844.1) using BWA-MEM (v0.7.18) [31] to assess genomic coverage and sequencing depth.

### 2.4. Statistical Analysis and Microbiome Data Visualization

Statistical analyses were performed in R (version 4.5.1) under a Linux environment using microbiome analysis packages, including vegan, phyloseq, ANCOMBC, and ALDEx2. Taxonomic abundance matrices were filtered and normalized according to individual analyses. Rarefaction was applied only for alpha-diversity estimation based on the minimum sequencing depth across samples, and rarefaction curves were generated to assess sequencing saturation. Alpha diversity was evaluated using Observed Features, Shannon diversity, and Pielou’s evenness indices. Differences between two groups were assessed using the Mann–Whitney U test, while multiple-group comparisons were performed using the Kruskal–Wallis H test with Bonferroni-adjusted post hoc tests.

Beta diversity was assessed by PCoA based on Bray–Curtis dissimilarities calculated from TSS-normalized abundance matrices. PERMANOVA (999 permutations) was used to evaluate differences in community composition, with R^2^ representing the explained variance. PERMDISP was performed to assess multivariate dispersion, and pairwise PERMANOVA comparisons were corrected using the Benjamini–Hochberg FDR method.

Differentially abundant taxa were screened using LEfSe (*p* < 0.05, LDA score > 3) and validated using ANCOM-BC2 and ALDEx2 [33,34]. Taxa with FDR < 0.05 in ANCOM-BC2 and ALDEx2 were considered significant. Only bacterial genera consistently identified by all three approaches were considered robust differential indicator taxa. Procrustes analysis and Mantel tests were conducted using Bray–Curtis distance matrices with 999 permutations to evaluate concordance between microbial communities and functional features.

To eliminate potential sample-size bias on network topological properties, the larger active group dataset was randomly subsampled to equalize the sample size with the quiescent group (*n* = 10) prior to the Spearman correlation analysis. Only robust correlations with an absolute coefficient (|r|) ≥ 0.9 and a false discovery rate (FDR)-adjusted *p*-value < 0.005 were retained as network edges. Following construction, network topological properties were calculated using the igraph R package [35], and the networks were visualized using Gephi(v0.11.2) [36]. To assess network complexity and identify keystone taxa, hub nodes were defined as those exhibiting a high degree (>25) and high betweenness centrality (>500) [37].

## 3. Results

### 3.1. Soil Metagenomes Revealed Highly Conserved Community Composition Across Four Natural Plague Foci

**Figure 1:**
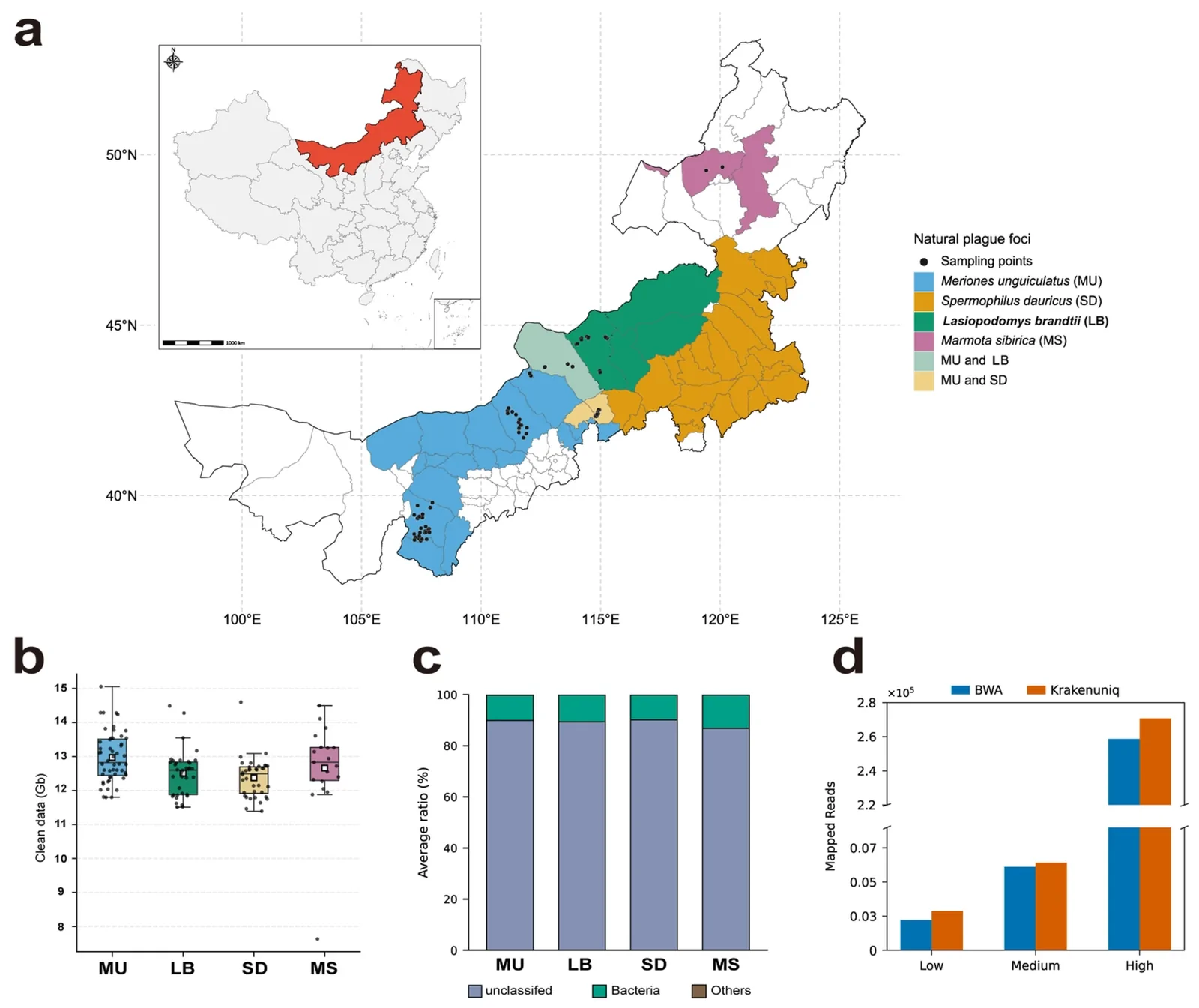
Metagenomic overview of soil microbiomes across four natural plague foci in Inner Mongolia. (**a**) Geographic distribution of soil sampling sites across four natural plague foci associated with *Meriones unguiculatus* (MU, *n* = 52), *Spermophilus dauricus* (SD, *n* = 36), *Lasiopodomys brandtii* (LB, *n* = 36), and *Marmota sibirica* (MS, *n* = 19). Colored regions indicate the spatial distribution of each plague focus, and black dots represent individual sampling locations. (**b**) Yield of clean metagenomic sequencing data (in Gb) across the soil samples from different foci. (**c**) Taxonomic composition of the soil microbial communities classified by KrakenUniq. (**d**) Concordance of metagenomic read assignments between KrakenUniq and BWA-MEM algorithms across different abundance tiers.

A total of 143 soil samples were collected from four natural plague foci in Inner Mongolia, encompassing habitats of *Meriones unguiculatus* (MU), *Lasiopodomys brandtii* (LB), *Spermophilus dauricus* (SD), and *Marmota sibirica* (MS) (Table S1; Figure 1a). To comprehensively characterize the soil microbiome, a hierarchical sampling strategy was implemented, incorporating surface and deep soil layers, plague-positive and control sites, and soils collected from inside (IB) and outside (OB) rodent burrows.

Shotgun metagenomic sequencing generated between 12.37 to 12.97 Gb of high-quality data per sample following quality filtering with Trimmomatic (Figure 1b). Taxonomic classification using KrakenUniq showed that 86.85–90.13% of reads remained unclassified, whereas classified reads were overwhelmingly dominated by bacteria (9.71–13.03%), with archaeal, eukaryotic, and viral sequences accounting for only minor fractions of the dataset (Figure 1c). Despite representing distinct host species and geographically separated plague foci, the four soil microbiomes exhibited remarkably similar community compositions at higher taxonomic ranks. To assess the robustness of taxonomic assignments, representative taxa spanning different abundance levels were independently validated by genome alignment using BWA. The strong concordance between KrakenUniq classification and read mapping supported the reliability of low-abundance taxa identified from the metagenomic data (Figure 1d).

Collectively, these results indicate that bacterial communities dominate the classifiable fraction of soil metagenomes across all plague foci while maintaining a highly conserved taxonomic framework, providing a robust basis for subsequent ecological comparisons.

### 3.2. Burrow-Associated Microbiomes Differed from Surrounding Soils Only Within the Quiescent Marmota sibirica Plague Focus

**Figure 2:**
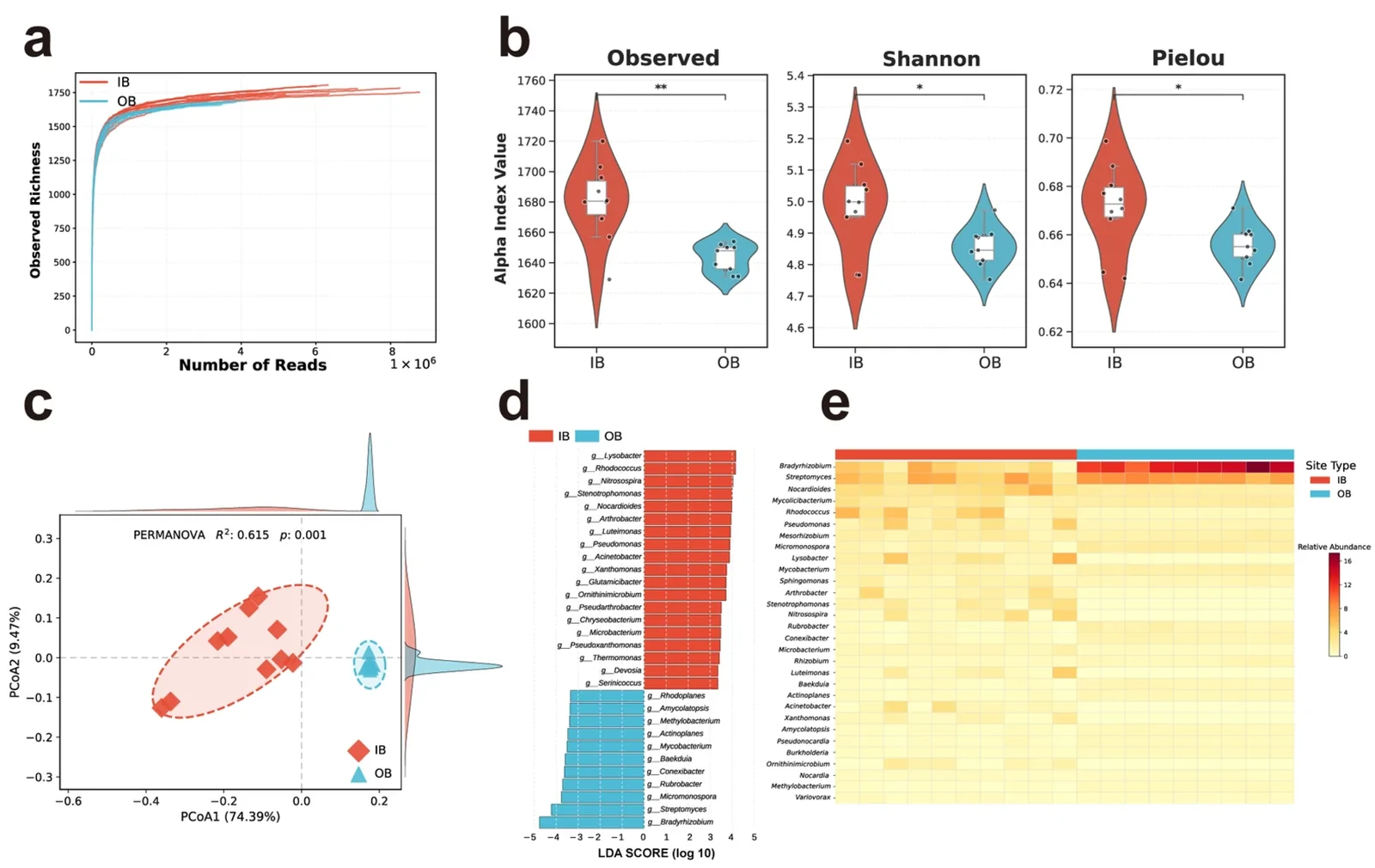
Microecological differentiation between inside-burrow (IB) and outside-burrow (OB) niches in the *Marmota sibirica* (MS) focus. (**a**) Genus-level rarefaction curves observed richness in IB and OB samples. (**b**) Genus-level α-diversity comparisons between IB and OB. * *p* < 0.05, ** *p* < 0.01. (**c**) PCoA based on Bray–Curtis dissimilarities (PERMANOVA); ellipses indicate 95% confidence intervals. (**d**) LEfSe-identified differentially abundant genera (LDA > 3). (**e**) Heatmap showing the relative abundances of the top 30 bacterial genera in the IB and OB soils.

To determine whether host burrows shape local microbial communities, soil samples collected from the IB and OB rodent burrows were compared independently within each plague focus (Table 1). No significant differences in either α- or β-diversity were observed between IB and OB soils in the MU, LB, or SD plague foci (Figure S1a–f). In contrast, the quiescent MS plague focus exhibited pronounced microbial community differentiation between the two microhabitats (*p* < 0.05; Figure 2b,c).

Within the MS dataset, rarefaction analysis indicated sufficient sequencing depth for all MS samples (Figure 2a). The IB soils displayed significantly greater microbial richness and community evenness than OB soils (*p* < 0.05; Figure 2b). Principal coordinate analysis (PCoA) revealed clear separation between IB and OB communities along the first axis (74.39% of variance explained), which was statistically supported by PERMANOVA (*R*^2^ = 0.615, *p* = 0.001; Figure 2c), alongside a significant difference in intra-group dispersion (PERMDISP, *p* = 0.001).

Although most bacterial taxa were shared between IB and OB soils (*n* = 228), IB communities contained a larger number of unique taxa (Figure S2a). Specifically, the UpSet plot reveals 74 taxa strictly unique to the IB microenvironment, compared to only 19 in the OB soils. Differential abundance analysis identified enrichment of *Lysobacter*, *Rhodococcus*, and *Nitrosospira* within burrows, whereas *Bradyrhizobium*, *Streptomyces*, and *Micromonospora* predominated outside burrows (*p* < 0.05; Figure 2d). Heatmap visualization confirmed that while *Bradyrhizobium* maintained a stable baseline abundance across both niches, the high abundance of *Streptomyces* was strictly localized to the OB environment (Figure 2e). Cross-validation using both ANCOM-BC2 and ALDEx2 consistently confirmed the enrichment of *Streptomyces* in OB soils (Figure S2b), supporting its robustness as a discriminatory taxon.

Within the MS plague focus, functional profiling revealed marked ecological differences between the two microhabitats. IB soils harbored greater diversity and richness of antimicrobial resistance genes (ARGs), including genes associated with tetracycline, β-lactam, and sulphonamide resistance (Figure S2c). Likewise, virulence-associated genes involved in adherence, regulation, nutrient acquisition, effector delivery, motility, invasion, and exotoxin production were significantly enriched within IB, whereas OB soils retained only a limited subset of regulatory functions (Figure S2d).

These observations suggest that rodent burrows constitute distinct ecological niches with enhanced microbial complexity and functional potential, particularly within the quiescent MS plague focus.

### 3.3. A Distinct Bacteriophage Community Characterizes the Quiescent MS Plague Focus

**Figure 3:**
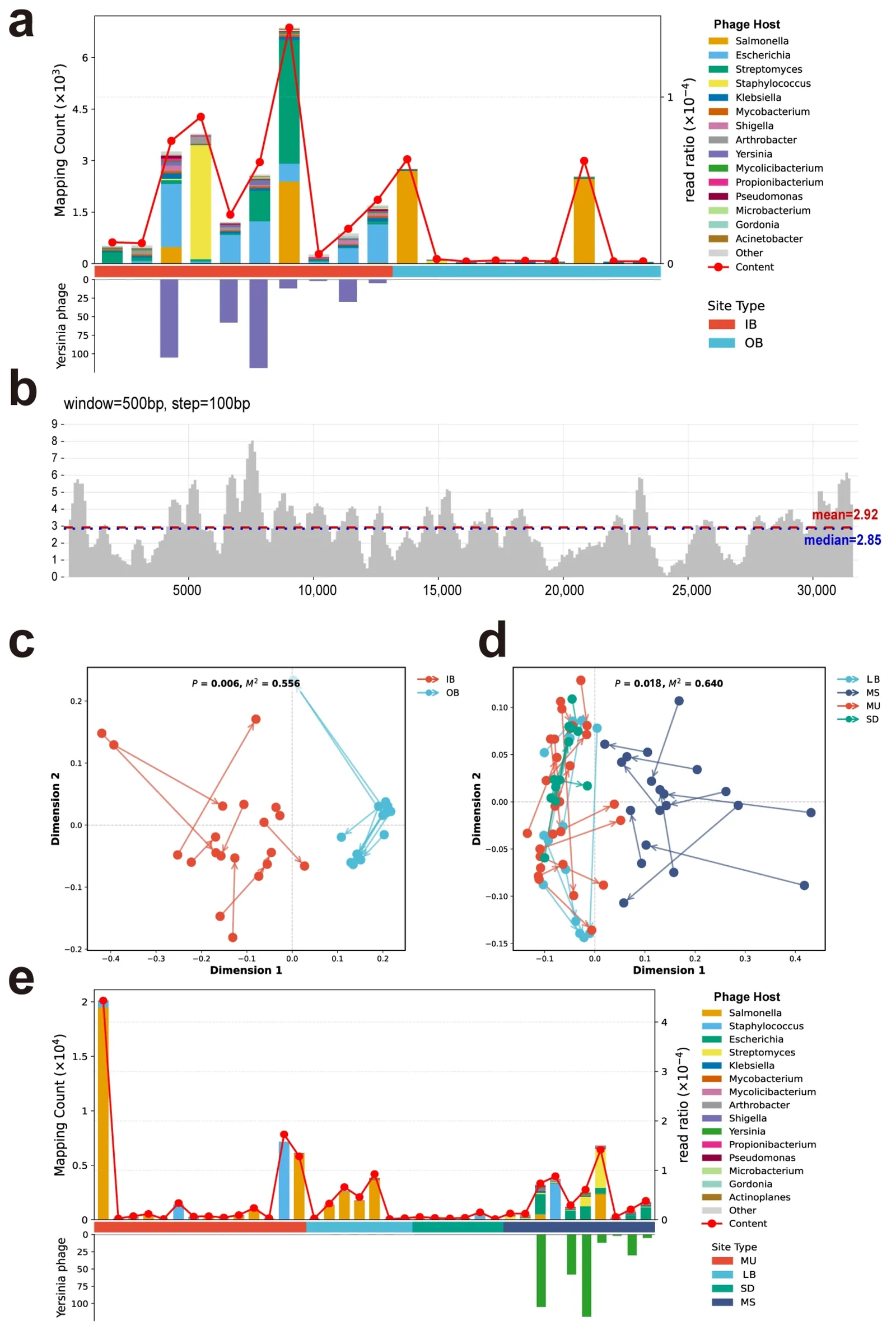
Soil bacteriophage communities and their ecological concordance with the bacterial community. (**a**,**e**) Taxonomic composition and richness of bacteriophages in the *Marmota sibirica* focus (**a**) and across all inside-burrow (IB) habitats (**e**). Stacked bars represent the BLAST-mapped reads of the 15 most abundant phage taxa, with the remainder grouped as ‘Other’. The red line denotes the cumulative proportion of mapped reads (right y-axis). (**b**) Genome coverage of *Yersinia* phage sequences detected in IB soils, mapped against the reference genome (NC_055844.1). Read depth was calculated via a sliding-window approach (window size = 500 bp). Red and blue lines indicate the mean and median mapping depths, respectively. (**c**,**d**) Procrustes analysis illustrating structural concordance between bacterial and bacteriophage communities within the *M. sibirica* focus (**c**) and across all IB habitats (**d**). Arrows connect paired communities from the same sample. The goodness-of-fit statistic (*M*^2^) and significance were determined via permutation testing.

Metagenomic bacteriophage profiling across all surveyed regions revealed that the spatial heterogeneity between IB and OB soils was markedly more pronounced in the MS plague focus compared to the MU, LB, and SD foci (Figure 3a). Bacteriophage diversity and relative abundance were consistently higher inside rodent burrows. IB bacteriophage communities were dominated by phages infecting *Streptomyces*, *Escherichia*, *Salmonella*, and *Yersinia*, whereas OB soils contained comparatively simpler bacteriophage communities largely composed of *Salmonella-* and *Klebsiella*-associated phages. Notably, reads homologous to *Yersinia* bacteriophages were detected exclusively in the IB soils and entirely absent from OB soils. Alignment against the temperate reference *Yersinia* phage genome (NC_055844.1) reconstructed 88.86% of the genome with an average sequencing depth of 2.93× and uniformly high mapping quality (MAPQ = 60), supporting the presence of closely related phage genomes within the soil metagenome (Figure 3b). However, it must be emphasized that these shotgun metagenomic readings indicate the existence of viral genomic DNA rather than active, live phage virions. Therefore, caution is advised when inferring active phage dynamics. Although these findings cannot distinguish active phage particles from residual environmental DNA, they provide metagenomic evidence for the persistence of *Yersinia*-associated phage sequences within the quiescent MS plague focus. Procrustes analysis further demonstrated strong concordance between bacterial and bacteriophage community structures (*M*^2^ = 0.556, *p* = 0.006; Figure 3c), suggesting a strong structural co-occurrence between bacteriophage and bacterial communities in burrow soils.

Subsequent profiling of bacteriophage communities in IB soils across the four plague foci revealed substantially greater phage diversity and richness in MS plague focus soils than in the other plague foci (Figure 3e). Bacteriophages associated with *Klebsiella* and *Salmonella* were widely distributed across all sites, whereas *Yersinia* bacteriophages were detected exclusively in the MS plague focus. Similarly, *Escherichia* phages were preferentially enriched in MS plague focus soils. Procrustes analysis demonstrated significant concordance between bacterial and bacteriophage community structures (*M*^2^ = 0.640, *p* = 0.018; Figure 3d).

### 3.4. Distinct Microbiome Configurations Distinguish the Four Natural Plague Foci

**Figure 4:**
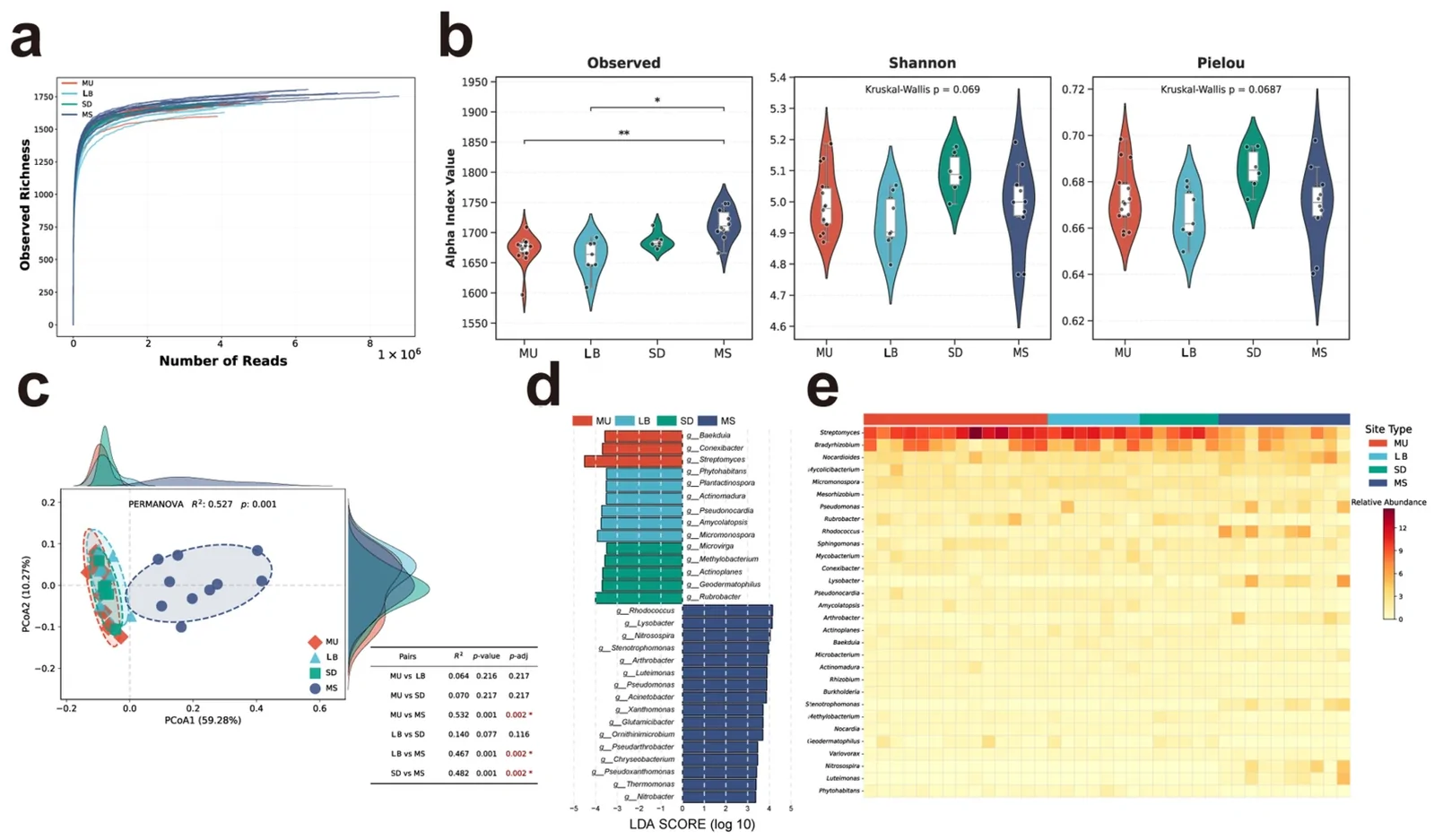
Ecological divergence of burrow-associated bacterial microbiomes across four natural plague foci. (**a**) Genus-level rarefaction curves observed bacterial richness. (**b**) Comparisons of genus-level α-diversity among the IB soil microbiomes. * *p* < 0.05, ** *p* < 0.01. (**c**) PCoA of bacterial community composition based on Bray–Curtis dissimilarities. (**d**) LEfSe-identified differentially abundant genera (LDA > 3). (**e**) Heatmap showing the relative abundances of the top 30 bacterial genera.

To investigate whether different natural plague foci harbor distinct soil microbiomes, we compared IB soil communities among the four host-associated plague foci (Table 1). Rarefaction analysis confirmed that sequencing depth was sufficient to capture bacterial diversity across all samples (Figure 4a). Alpha-diversity analysis revealed comparable Shannon diversity and Pielou’s evenness among the four groups, whereas microbial richness was significantly greater in the MS plague focus than in the MU and LB foci (*p* < 0.05; Figure 4b). PCoA analysis based on Bray–Curtis dissimilarities demonstrated clear separation of microbial communities among plague foci, with the first principal coordinate (59.28% of explained variation) primarily distinguishing samples from the MS plague focus (Figure 4c). PERMANOVA confirmed significant differences in community composition among the four foci (*R*^2^ = 0.527, *p* = 0.001), accompanied by significant variations in intra-group dispersion (PERMDISP, *p* = 0.008).

Despite a large proportion of shared core microbiomes across all four IB habitats, the MS focus exhibited the highest taxonomic exclusivity (Figure S3a). LEfSe analysis identified *Streptomyces* and *Conexibacter* as indicator taxa of the MU focus, *Micromonospora* and *Amycolatopsis* of the LB focus, and *Rubrobacter* together with *Geodermatophilus* of the SD focus (*p* < 0.05; Figure 4d). In contrast, the MS focus was uniquely enriched for *Rhodococcus* and *Lysobacter*. Strikingly, the relative abundance of *Streptomyces* was severely depleted in MS soils compared to the other foci, whereas *Bradyrhizobium* maintained a stable baseline across all groups (Figure 4e).

Functional profiling of ARGs and VFs further demonstrated distinct ecological characteristics of the MS plague focus microbiome. Whereas ARG profiles in the MU, LB, and SD foci were dominated by macrolide resistance determinants, tetracycline resistance genes predominated within the MS plague focus microbiome (Figure S3b). Similarly, virulence profiles in MU, LB, and SD plague foci were largely restricted to regulatory traits with low overall VF richness, whereas the MS plague focus displayed a comprehensive enrichment across a broad spectrum of virulence categories (Figure S3c).

Collectively, these analyses indicate that the quiescent MS plague focus harbors a distinct bacterial and functional gene profile relative to the three epidemiologically active plague foci, highlighting a microbiome configuration unique to this specific host and geographic setting.

### 3.5. Burrow-Associated Soil Provides the Greatest Ecological Resolution for Microbiome-Based Surveillance

To evaluate how sampling strategy influences microbiome characterization, we compared metagenomes generated from three soil matrices, including surface soil, deep soil, and IB soil (Table S2). Neither surface nor deep soil exhibited significant differences in microbial α-diversity or community composition between plague-positive and control sites (*p* > 0.05; Figure S4a and Figure 4b), with no significant differences in intra-group dispersion (PERMDISP, *p* > 0.05). In contrast, burrow-associated soils exhibited greater ecological differentiation. Samples collected from plague-positive burrows displayed significantly higher microbial richness and a distinct overall community composition (PERMANOVA, *R*^2^ = 0.067, *p* = 0.003; Figure S4c) compared to control burrows. Community dispersion increased markedly within plague-positive burrow soils (PERMDISP, *p* = 0.032), indicating pronounced spatial heterogeneity associated with the burrow microenvironment. Furthermore, burrow-associated soils consistently contained richer functional gene repertoires than either surface or deep soils (Figure S4d). Specifically, phage richness in burrow microbiomes was significantly higher than in both surface and deep soils (*p* < 0.05). Additionally, burrow soil contained significantly higher ARG richness than surface soil, and significantly higher VF richness than both surface and deep soils (all *p* < 0.05).

These findings indicate that burrow-associated soil preserves substantially greater taxonomic and functional information than conventional soil sampling approaches, suggesting its potential value as a sensitive substrate for future microbiome-based ecological monitoring in natural plague foci.

### 3.6. Soil Microbiome Composition Differs Between Epidemiologically Active and Quiescent M. sibirica Focus

To identify microbiome characteristics associated with plague epidemiological status, burrow-associated soils were grouped according to the epidemiologically active (MU, LB, and SD) or the quiescent (MS) foci (Table S3). At the phylum level (Figure S5a), the active foci were predominantly dominated by Actinomycetota (53.10%) and Pseudomonadota (38.01%), whereas the quiescent MS focus exhibited a reversed dominance of Pseudomonadota (47.08%) and Actinomycetota (45.42%), alongside an enriched proportion of Bacteroidota (3.08% vs. 0.76%). At the genus level, active foci were heavily enriched with *Streptomyces* (11.59%), *Bradyrhizobium* (6.27%), and *Nocardioides* (3.31%). Conversely, the quiescent MS focus displayed a more evenly distributed dominant community, marked by elevated relative abundances of *Rhodococcus* (3.92%), *Lysobacter* (3.46%), and *Pseudomonas* (3.18%).

**Figure 5:**
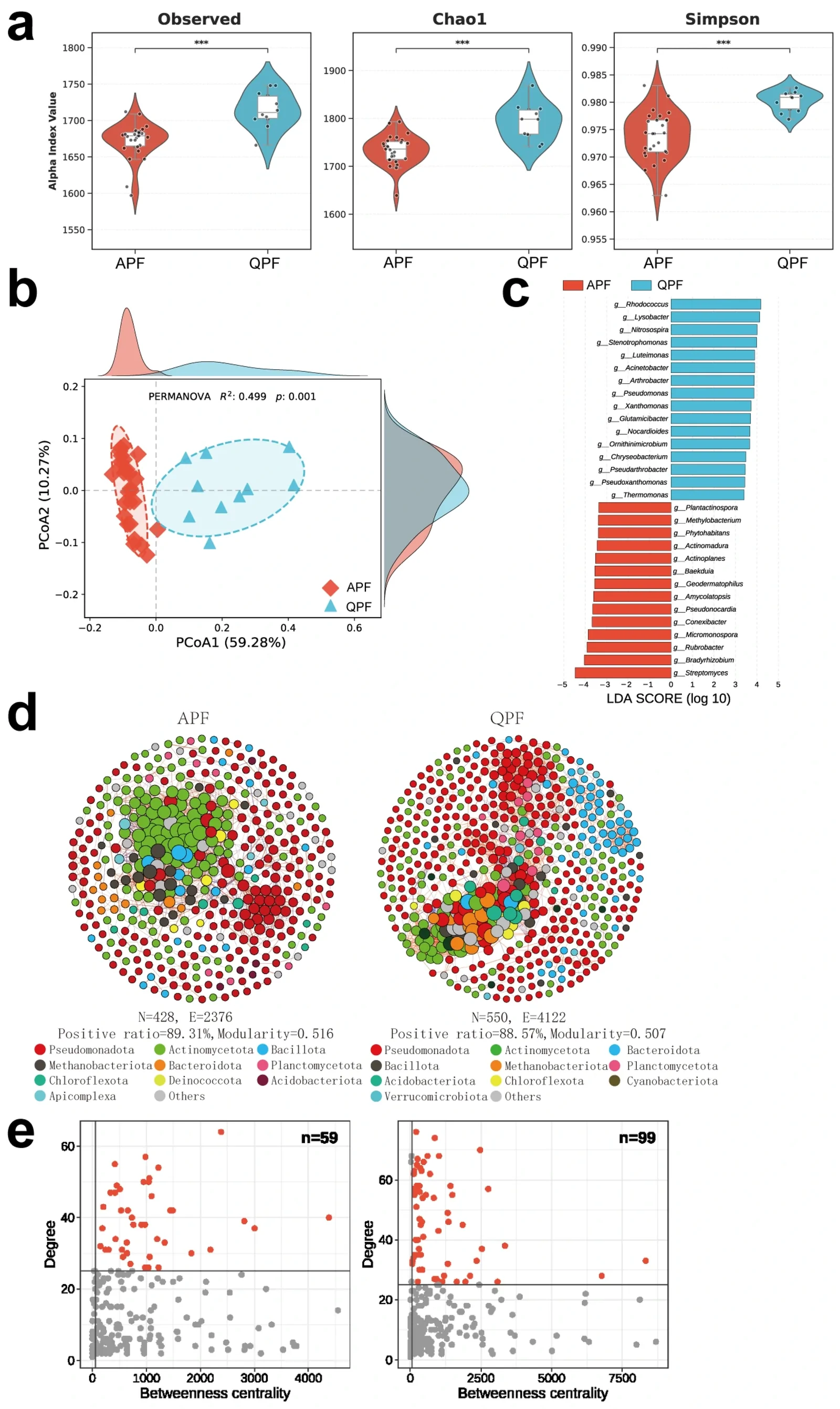
Community diversity, structure, and co-occurrence networks of burrow soil microbiomes in active (APF) and quiescent (QPF) plague foci. (**a**) Comparisons of α-diversity between the IB soil microbiomes from active and quiescent plague foci. *** *p* < 0.001. (**b**) PCoA based on Bray–Curtis dissimilarities showing separation of the genus-level bacterial communities between active and quiescent plague foci. (**c**) LEfSe analysis identifying significant genus-level indicator taxa in active (red) and quiescent (blue) soils. (**d**) Co-occurrence networks colored by phylum. (**e**) Identification of keystone hub nodes (red) based on node degree and betweenness centrality.

The quiescent MS focus exhibited significantly greater microbial richness than the three active plague foci (*p* < 0.001; Figure 5a). PCoA analysis further demonstrated clear separation between the two groups along the first principal coordinate, accounting for 59.28% of the total variation (Figure 5b). PERMANOVA confirmed that this grouping (active foci vs. quiescent MS focus) explained a significant proportion of the observed community variation (*R*^2^ = 0.499, *p* = 0.001), along with a significant difference in intra-group dispersion (PERMDISP, *p* = 0.005).

To identify the specific taxonomic drivers associated with these groups, LEfSe analysis revealed that *Streptomyces* exhibited the highest discriminative power in active foci. In contrast, the quiescent MS focus was characterized by a significant enrichment of genera such as *Rhodococcus*, *Lysobacter*, *Nitrosospira*, and *Stenotrophomonas* (*p* < 0.05; Figure 5c). A heatmap of the top 30 genera visually corroborated this pattern, highlighting the persistently high abundance of *Streptomyces* in active foci and its stark depletion in the quiescent MS focus (Figure S5b).

Finally, we constructed co-occurrence networks to evaluate systemic ecological interactions (Figure 5d and Table S4). Active foci microbiomes formed a relatively simple network comprising 428 nodes and 2376 edges, with a modularity of 0.516. In stark contrast, the quiescent MS focus maintained a substantially larger and more densely connected network encompassing 550 nodes and 4122 edges, with a modularity of 0.507. Furthermore, by jointly evaluating node degree and betweenness centrality, we identified only 59 hub nodes in the active foci network compared to 99 hub nodes in the quiescent MS focus (Figure 5e).

These analyses identified a consistent microbiome separation between the three epidemiologically active foci and the quiescent MS focus, although this divergence likely reflects a combination of epidemiological status, host species, and localized geographic features.

## 4. Discussion

The ecological persistence of plague has traditionally been explained by interactions among *Yersinia pestis*, rodent hosts, flea vectors, and environmental factors [38]. However, the surrounding soil microbiome has received comparatively little attention despite representing the immediate ecological context of pathogen transmission [39]. Here, by integrating bacterial, bacteriophage, and functional metagenomic profiles, we demonstrate that soil microbial communities exhibit pronounced heterogeneity across distinct natural plague foci in Inner Mongolia. More importantly, from an epidemiological perspective, these findings reveal coordinated microbiome divergences when comparing the active plague foci to the quiescent MS focus. Rather than treating the belowground environment as a passive background, our findings suggest that soil microbial communities display potential associations with long-term epidemiological heterogeneity and should be considered an integral ecological component of natural plague foci.

The most distinctive microbiome was observed in the epidemiologically quiescent MS plague focus, which exhibited higher microbial richness together with coordinated shifts in bacterial, bacteriophage, and functional gene composition. Our network analysis further demonstrated that this quiescent MS focus harbors a highly complex and interconnected microbial co-occurrence network. While recent studies [39] suggesting that higher community diversity and topological complexity may be associated with ecosystem stability and resistance to ecological perturbation, it must be noted that correlation-based networks represent statistical co-occurrences rather than verified biological interactions. Therefore, inferences regarding enhanced ecosystem stability or resilience in the quiescent focus remain speculative and should be interpreted with caution. Conversely, active plague foci were characterized by significantly lower microbial richness, simplified network structures, and the consistent enrichment of *Streptomyces*, *Bradyrhizobium* and *Rubrobacter*. Members of the genus *Streptomyces* are widely distributed in soil ecosystems and are renowned for their vast biosynthetic capacity to produce antimicrobial agents [40,41,42]. In our dataset, the high abundance of these potential secondary metabolite producers inversely correlates with the overall microbial richness and network complexity in active foci. While this inverse relationship may suggest potential competitive dynamics or overlapping environmental preferences between *Streptomyces* and indigenous taxa, current metagenomic profiles cannot definitively prove direct *Streptomyces*-driven competitive exclusion. Future in vitro co-culture experiments are required to elucidate whether these enriched taxa actively shape the local ecological niche or merely co-occur with *Y. pestis* in chemically or ecologically destabilized environments.

A notable finding was the exclusive detection of *Yersinia*-associated bacteriophage sequences in the quiescent MS plague focus, accompanied by significant concordance between bacterial and bacteriophage community structures. Bacteriophages are increasingly recognized as major regulators of bacterial community dynamics [43,44], providing theoretical frameworks for understanding these interactions. However, our findings remain purely observational and must be interpreted cautiously. Metagenomic sequencing cannot distinguish active phage infection from residual environmental DNA [45,46,47]. Crucially, as no phages were isolated and no infectivity assays were performed in this study, there is no direct evidence linking these temperate phages to the active infection or dynamic regulation of *Y. pestis* populations. Therefore, we do not imply a functional role for these phages in maintaining plague quiescence. Instead, our results reveal a previously unrecognized ecological association between *Yersinia*-related phage signatures and this specific *M. sibirica* microenvironment, highlighting potential localized ecological biomarkers that merit further experimental investigation to disentangle host- and location-specific confounding variables from broad epidemiological quiescence. Unlike broad genera like *Streptomyces*, the strict host specificity of *Yersinia* bacteriophages makes them a uniquely reliable biomarker for tracing the pathogen’s ecological footprint. Furthermore, even when *Y. pestis* is absent, these temperate phages persist—likely via lysogenic integration into rare hosts or DNA adsorption to soil matrices—serving as an enduring historical footprint of past pathogen circulation.

Alongside taxonomic shifts, the significant enrichment of virulence-associated genes in the quiescent MS focus—particularly for regulation and effector delivery—aligns closely with established *Yersinia* mechanisms. Notable examples include Caf1R-mediated regulation of the F1 capsular antigen [48] and the LcrV/LcrG-regulated type III secretion system (T3SS) [49]. Although metagenomics cannot confirm active expression, the accumulation of these genetic elements suggests that burrow microenvironments serve as significant ecological reservoirs for complex virulence determinants.

Moreover, long-term environmental persistence requires sophisticated colonization and stress-adaptation mechanisms. Key detected features, including chaperone-usher fimbriae [50] and the Cpx/RovM signaling cascade governing *Yersinia* biofilm formation [51,52], facilitate microbial survival under abiotic stress. Such stressors also drive the resistome [53,54], where the widespread antimicrobial resistance genes (ARGs) we observed are likely maintained via horizontal gene transfer driven by mobile plasmids and conjugative pili [55,56]. Ultimately, while our profiling highlights how environmental stress shapes microbiome resilience [57], future multi-omics studies are needed to fully elucidate these intricate ecosystem dynamics [58].

Beyond advancing our understanding of plague ecology, this study has implications for One Health surveillance [38,59]. Current plague surveillance primarily relies on monitoring rodents, fleas, and pathogen isolation [38], whereas microbiome-based environmental surveillance remains largely unexplored. We found that burrow-associated soils retained greater bacterial diversity, richer functional gene repertoires, and substantially higher bacteriophage diversity than either surface or deep soils, indicating that they preserve stronger ecological signals of plague habitats. Consequently, prioritizing the collection of burrow-associated soils in future field investigations could optimize research efficiency and significantly reduce sampling costs. Integrating such targeted soil microbiome profiling with conventional wildlife surveillance holds potential to provide complementary ecological indicators for monitoring plague-endemic ecosystems. However, as our current cross-sectional data do not provide direct empirical evidence for improved plague detection or forecasting, future longitudinal investigations are necessary to validate the predictive capabilities of these environmental indicators.

Several limitations of this study should be acknowledged. First, our sampling design included only a single quiescent focus (the *M. sibirica* habitat) against three active foci, resulting in unequal sample sizes across the compared groups. Consequently, the epidemiological status (active vs. quiescent) in our analysis is inherently confounded by host species, geographic location, host-specific burrow ecology, and underlying soil type, and potential statistical biases introduced by unbalanced sampling. It remains impossible to definitively determine whether the observed differences are driven strictly by plague quiescence or by the specific ecological and geographic niche of the *M. sibirica* burrows. Therefore, the variations observed in our study must be interpreted as ecological associations rather than causal relationships. Additionally, the cross-sectional nature of our study precludes the assessment of temporal dynamics. Finally, shotgun metagenomics provides information on community composition and functional potential but not microbial activity or viability. Furthermore, taxonomic profiling using KrakenUniq resulted in a high proportion of unclassified reads, a phenomenon common in complex soil metagenomes due to the underrepresentation of environmental microbes in standard reference databases. Consequently, this reliance on known databases limits the sensitivity for discovering novel, uncharacterized, or highly divergent low-abundance taxa in these plague foci.

A further critical limitation of our current study is the absence of environmental and soil physicochemical metadata, such as soil pH, moisture content, organic carbon, and texture. It is well established that these abiotic factors play a pivotal role in shaping soil microbial assembly, diversity, and functional potentials. For instance, soil chemistry can strongly influence microbial interactions and taxonomic enrichments. Without accounting for these baseline characteristics, we cannot confidently distinguish whether key observations (e.g., the pronounced *Streptomyces* depletion) are driven by plague-related ecological shifts (quiescence) or if they merely reflect baseline differences in local soil chemistry. Additionally, from a statistical perspective, the significant dispersion effects (PERMDISP) observed across our datasets indicate that group heterogeneity also contributes to the PERMANOVA outcomes. Thus, these microbiome variations must be interpreted as a combination of compositional shifts and varying degrees of spatial dispersion, rather than absolute community replacement. Future studies integrating longitudinal sampling, robust environmental metadata collection, and in vitro experimental validation will be essential to determine whether soil microbiomes merely reflect, or actively influence, the long-term persistence of natural plague foci.

Overall, our study expands the current understanding of plague ecology by incorporating the environmental microbiome into the conceptual framework of natural plague foci. Rather than serving solely as a background habitat, soil microbial communities may represent ecological indicators associated with long-term plague persistence and quiescence. These findings provide a foundation for microbiome-informed environmental surveillance and broaden the application of metagenomics in the ecological monitoring of zoonotic diseases.

## 5. Conclusions

This study revealed that soil microbial communities and their functional potentials exhibit pronounced heterogeneity across distinct natural plague foci. The burrow-associated microbiomes in the quiescent MS focus, particularly their bacteriophage assemblages and network keystone taxa, exhibited highly distinct profiles characterized by greater microbial diversity, heightened topological complexity, an accumulation of specific virulence and antimicrobial resistance genes, and the exclusive presence of *Yersinia*-associated phage signatures. In contrast, microbiomes in the three active plague foci displayed simplified co-occurrence networks alongside a pronounced enrichment of potential competitors, such as *Streptomyces*. Our multi-dimensional analyses indicate that these structural and topological variations are associated with these distinct focal types, although future studies must disentangle broad epidemiological patterns from host- and location-specific confounding variables, such as the localized ecological niche and underlying soil chemistry of the *M. sibirica* habitat. Overall, this work identifies specific soil microbiome signatures—including taxonomic compositional shifts, varied network topological complexity, the persistence of *Yersinia* bacteriophages, and functional gene enrichment—that are intimately associated with the microecological landscapes of plague foci. This provides a data-driven basis for integrating belowground microbiome profiling into One Health surveillance strategies in plague-endemic regions.

## Figures and Tables

**Table 1:** Distribution and sampling characteristics of burrow-associated soils across plague foci.

Plague Foci	Inside Burrow	Outside Burrow	Region
*Meriones unguiculatus*	14	8	Erenhot City, Sonid Left, Siziwang Banner
*Lasiopodomys brandtii*	7	5	Abaga Banner
*Spermophilus dauricus*	6	6	Zhengxiangbai Banner
*Marmota sibirica*	10	9	Chen Barag Banner

## Data Availability

The datasets generated and/or analyzed during the current study are available in the NCBI Sequence Read Archive (SRA) under BioProject accession number PRJNA1494090 (https://www.ncbi.nlm.nih.gov/bioproject/PRJNA1494090, accessed on 5 August 2026).

## Supplementary Materials

The following supporting information can be downloaded at: https://www.mdpi.com/article/10.3390/microorganisms14102329/s1, Figure S1: Diversity of IB and OB soil microbiomes in the MU, LB, and SD foci. Figure S2: Taxonomic overlap and functional genetic profiles of the IB and OB microbiomes in the MS focus. Figure S3: Taxonomic conservation and functional divergence of IB microbiomes across the four plague foci. Figure S4: Microbial community divergence and functional genetic element richness across distinct soil sampling substrates. Figure S5: Taxonomic profiles across active (APF) and quiescent (QPF) plague foci. Table S1: Sampling details and geographic distribution of soils across natural plague foci in Inner Mongolia. Table S2: Comparison of soil sampling strategies across natural plague foci. Table S3: Sampling characteristics and diversity metrics of IB soils across active and quiescent plague foci. Table S4: Topological properties of microbial co-occurrence networks in active and quiescent plague foci.
