# Supplementary material for: Soil Microbiome Signatures Associated with Epidemiological Heterogeneity Across Natural Plague Foci in Inner Mongolia, China
Source: Microorganisms. 2026 Oct 13;14(10):2329. doi: 10.3390/microorganisms14102329 (PMC13707690; doi:10.3390/microorganisms14102329)
Supplement: Supplementary file 1 [file microorganisms-14-02329-s001.zip › microorganisms-4594105-supplementary.pdf]

Supplementary Materials

Supplementary Tables

Table S1. Sampling details and geographic distribution of soils across natural plague foci in Inner Mongolia.

Plague foci	Surface soil	Deep soil	Inside Burrow	Outside Burrow	Region	Epidemiological state
Meriones unguiculatus	15/15	0	14	8	Otog, Sonid Left, Siziwang Banner, Erenhot City	Active
Lasiopodomys brandtii	7/5	7/5	7	5	Abaga Banner	Active
Spermophilus dauricus	6/6	6/6	6	6	Zhengxiangbai Banner	Active
Marmota sibirica	0	0	10	9	Chen Barag Banner	Quiescent

Note: Values are presented as X/Y, indicating the number of samples collected from plague-positive and control sites within the plague focus, respectively.

Table S2. Comparison of soil sampling strategies across natural plague foci.

Group	Plague	Control
Surface soil	28	26
Deep soil	13	11
Burrow soil	37	28

Table S3. Sampling characteristics and diversity metrics of IB soils across active and quiescent plague foci.

Group	N	α-diversity			β-diversity	
		Observed	Chao1	Simpson	R^2	P-value
APF	27	1672.15 ± 24.8	1733.76 ± 31.38	0.97 ± 0.00	0.499	0.001
QPF	10	1714.3 ± 25.76	1794.05 ± 39.27	0.98 ± 0.00		

Table S4. Topological properties of microbial co-occurrence networks in active and quiescent plague foci.

Network topological properties	APF	QPF
Nodes	428	550
Edges	2376	4122
Positive edges (%)	89.31	88.57
Negative edges (%)	10.69	11.43
Average degree	11.103	14.989
Average path length	4.954	5.204
Modularity	0.516	0.507
Graph density	0.026	0.027

Supplementary Figures

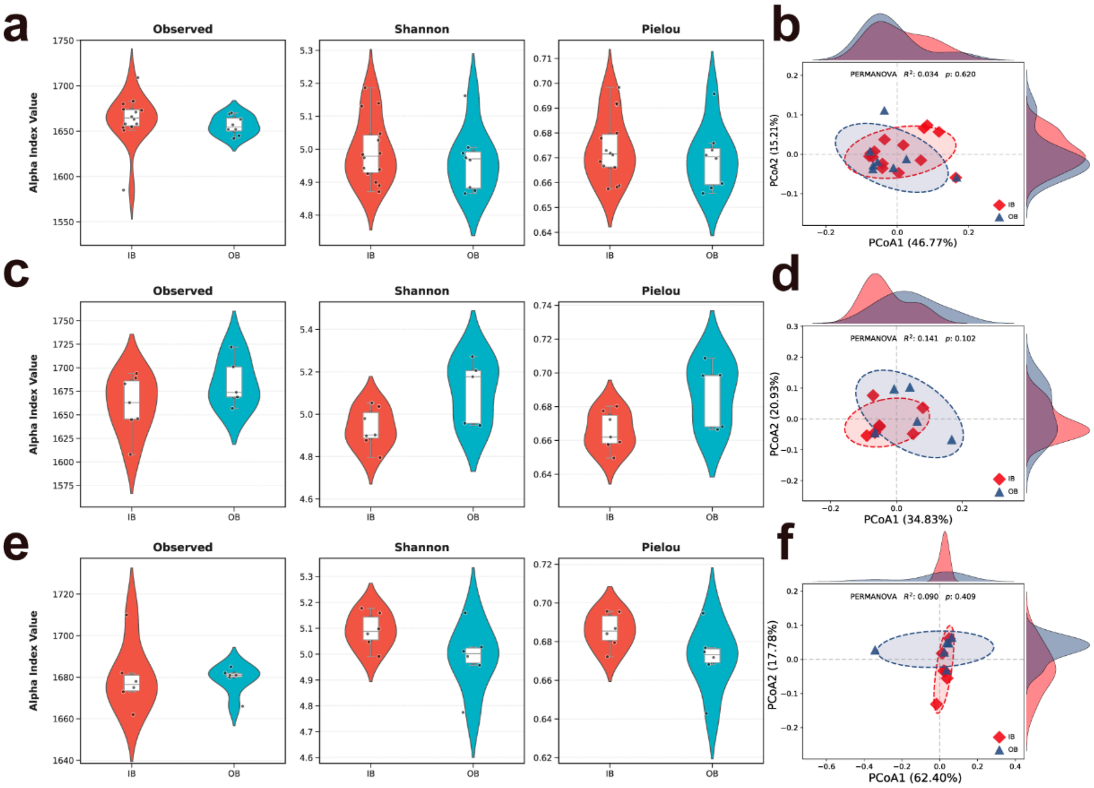

Figure S1. Diversity of IB and OB soil microbiomes in the MU, LB, and SD foci. **(a, c, e)** Genus-level α-diversity comparisons between the IB and OB soil samples in the MU **(a)**, LB **(c)**, and SD **(e)** foci. **(b, d, f)** PCoA based on Bray–Curtis dissimilarities showing separation of the genus-level bacterial communities between IB and OB soils in the MU **(b)**, LB **(d)**, and SD **(f)** foci.

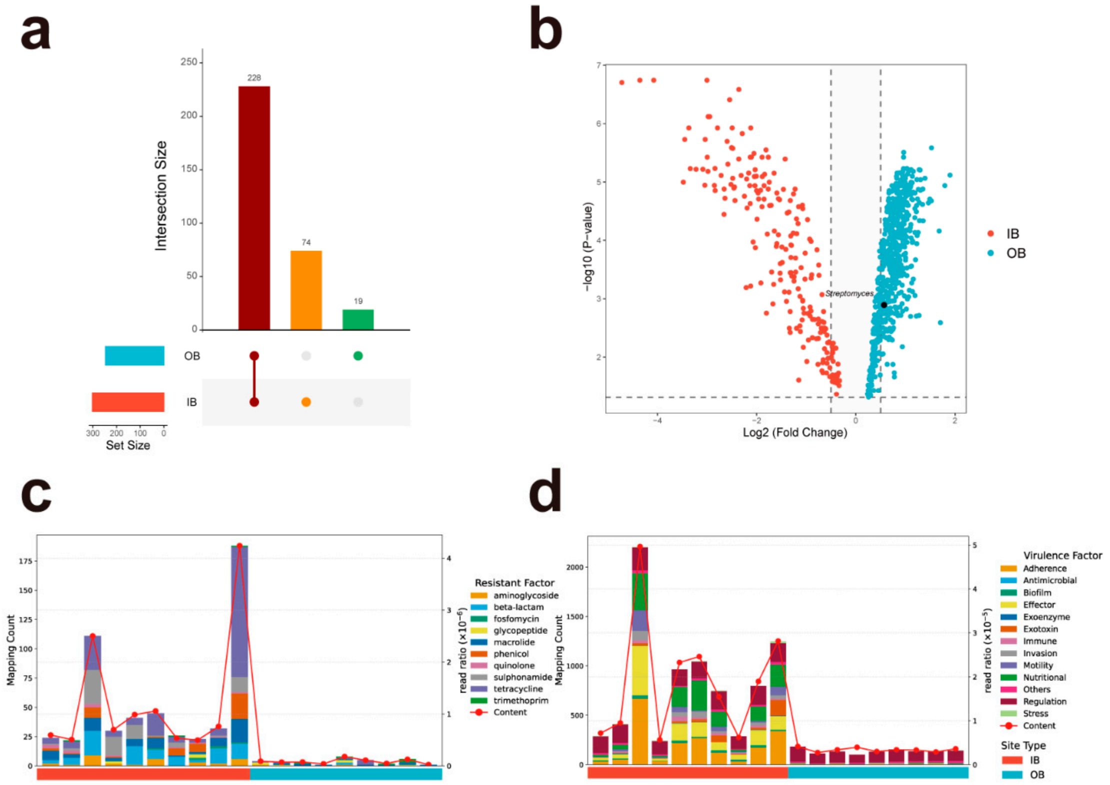

Figure S2. Taxonomic overlap and functional genetic profiles of the IB and OB microbiomes in the MS focus.

(a) UpSet plot showing the shared and unique bacterial genera between the IB and OB soils. Connected dots represent taxa shared across habitats, whereas single, unconnected dots denote site-specific unique taxa restricted to either IB or OB soils. **(b)** Volcano plot showing differentially abundant bacterial genera identified by ANCOM-BC2 and validated by ALDEx2. Dashed lines indicate the effect size and significance thresholds (|$\log_2$ fold change| = 0.5; adjusted $P = 0.05$). The black point highlights *Streptomyces* enrichment in the OB microenvironment. **(c, d)** Composition and abundance of antimicrobial resistance genes (ARGs) **(c)** and virulence factors (VFs) **(d)** across IB and OB niches, showing BLAST alignment counts and cumulative read proportions.

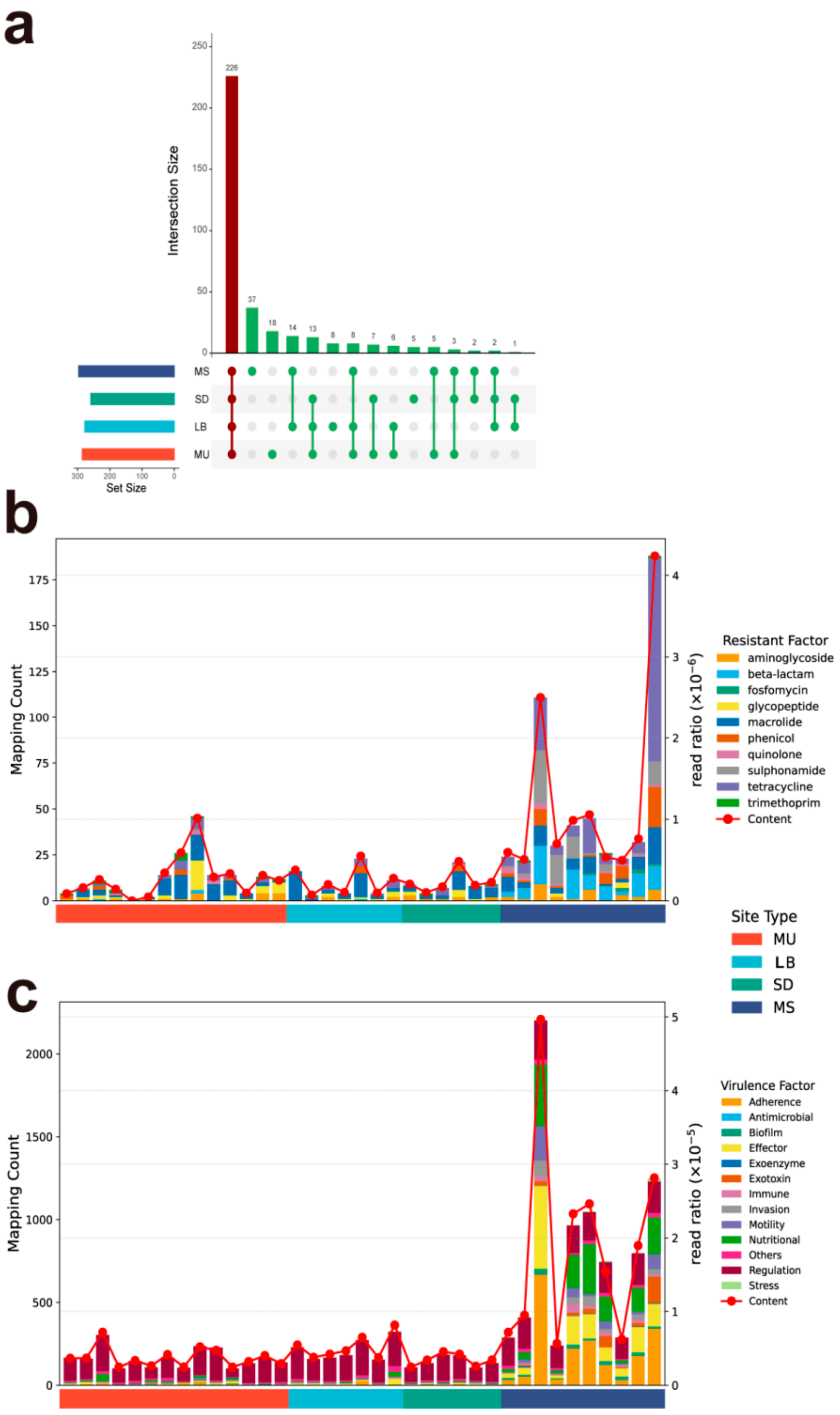

Figure S3. Taxonomic conservation and functional divergence of IB microbiomes across the four plague foci.

(a) UpSet plot showing the shared and unique bacterial genera among the IB soils of the MU, LB, SD, and MS foci. **(b, c)** Composition and abundance of antimicrobial resistance genes (ARGs; **b**) and virulence factors (VFs; **c**) in IB soils across four plague foci.

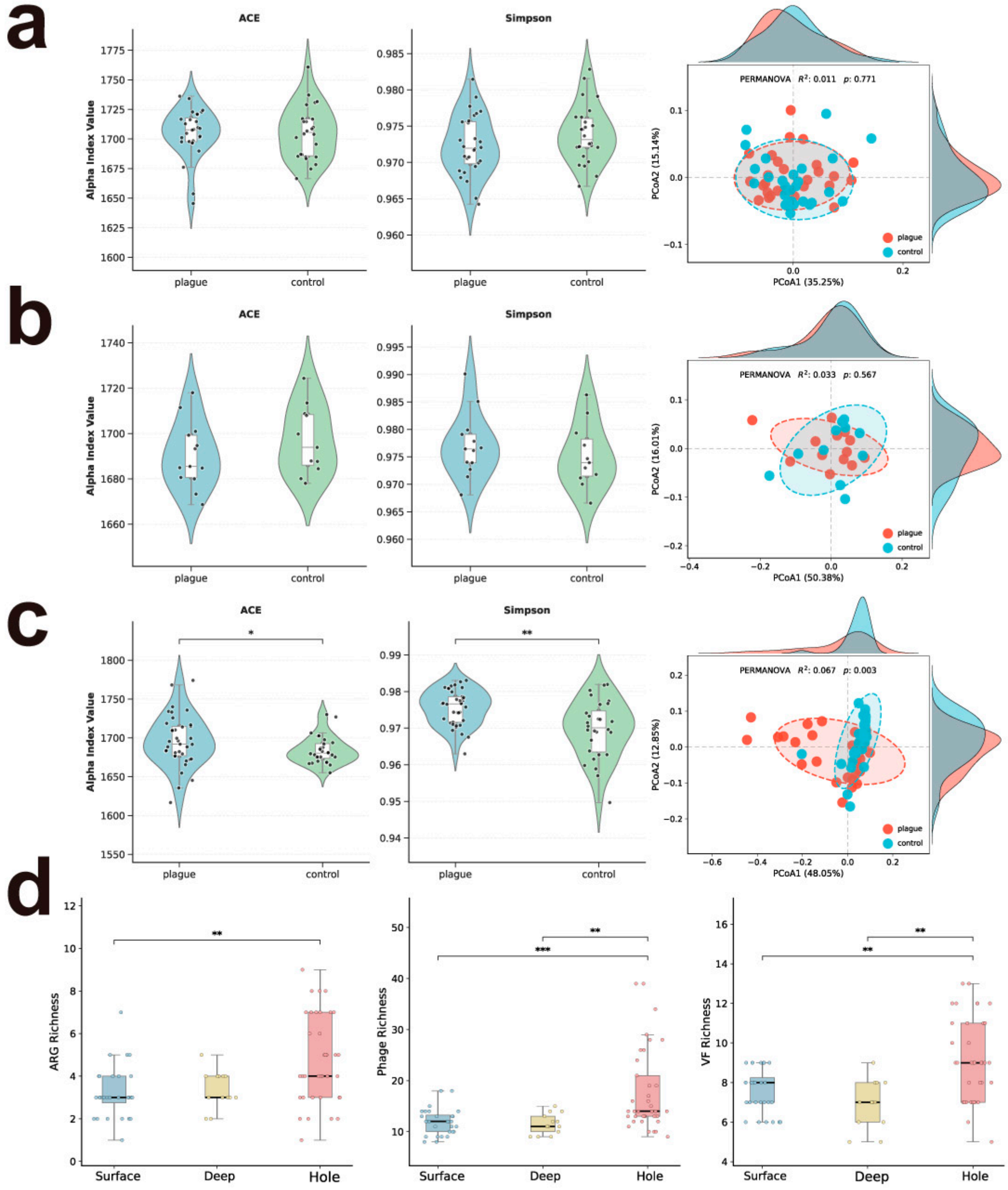

Figure S4. Microbial community divergence and functional genetic element richness across distinct soil sampling substrates.
(a–c) Comparisons of genus-level α-diversity and principal coordinate analysis (PCoA) based on Bray–Curtis dissimilarities in surface **(a)**, deep **(b)**, and burrow **(c)** soils from the plague and control groups. **(d)** Richness of antimicrobial resistance genes (ARGs), phages, and virulence factors (VFs) across the three soil substrates within the plague foci. *$p < 0.05$, **$p < 0.01$, ***$p < 0.001$.

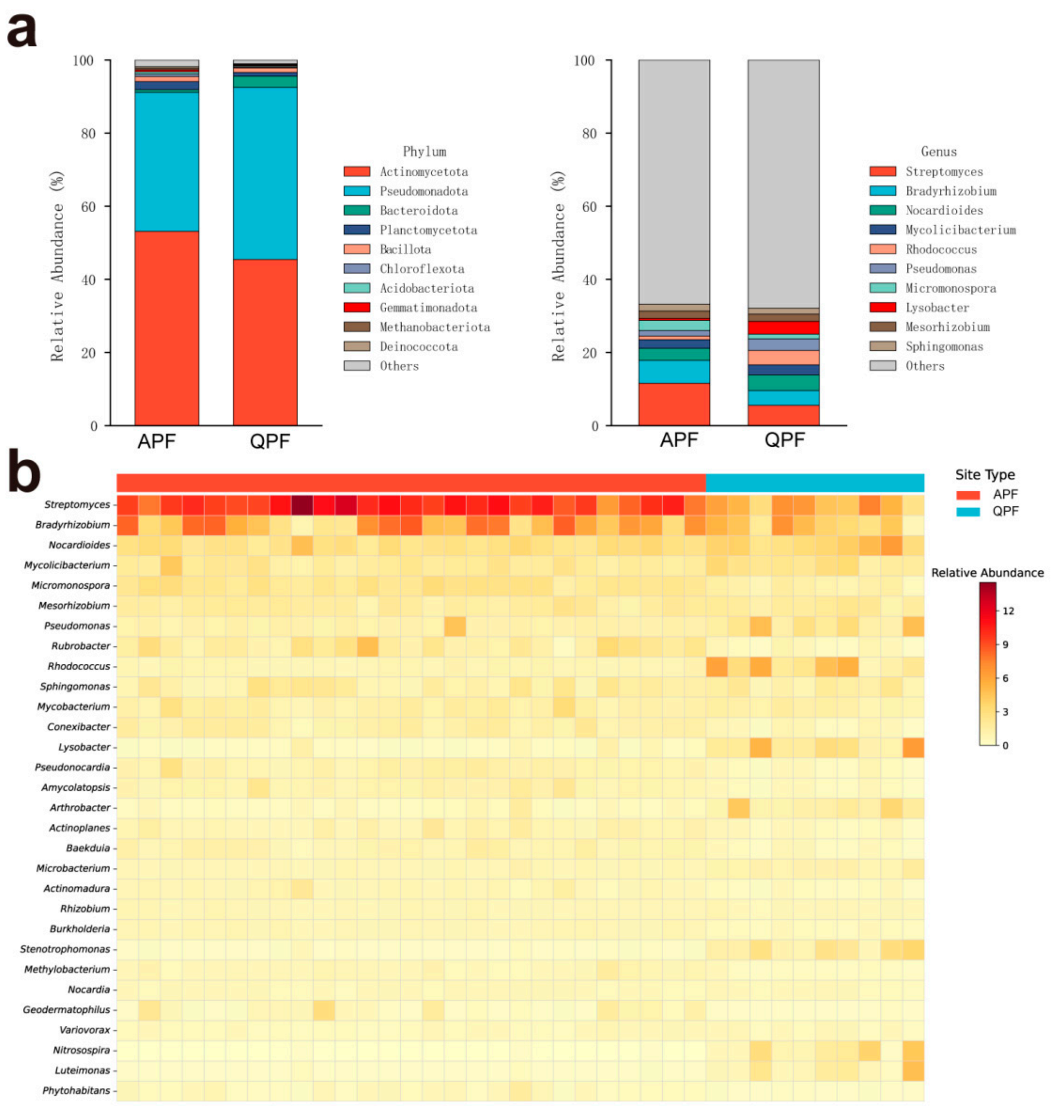

Figure S5. Taxonomic profiles across active (APF) and quiescent (QPF) plague foci.
(a) Relative abundances of the top 10 bacterial phyla and genera. **(b)** Heatmap showing the relative abundances of the top 30 bacterial genera in the active and quiescent plague foci.
